# The relationship between the modified frailty index score (mFI-5), malnutrition, body composition, systemic inflammation and short-term clinical outcomes in patients undergoing surgery for colorectal cancer

**DOI:** 10.1186/s12877-022-03703-2

**Published:** 2023-01-06

**Authors:** Josh McGovern, Alexander Grayston, Dominic Coates, Stephen Leadbitter, Adam Hounat, Paul G. Horgan, Ross D. Dolan, Donald C McMillan

**Affiliations:** Academic Unit of Surgery, School of Medicine, University of Glasgow, Glasgow Royal Infirmary, Level 2, New Lister Building, G31 2ER Glasgow, UK

**Keywords:** Frailty, mFI-5, Short-term outcomes, Inflammation

## Abstract

**Background:**

While the current literature suggests an association with frailty and clinical outcomes in patients undergoing surgery for colorectal cancer (CRC), the basis of this relationship is unclear.

**Aim:**

Examine the relationship between frailty, malnutrition, body composition, systemic inflammation and short-term clinical outcomes in patients undergoing surgery for colorectal cancer.

**Methods:**

Consecutive patients who underwent potentially curative resection for colorectal cancer, between April 2008 and April 2018, were identified from a prospectively maintained database. Frailty was defined using the modified five-item frailty index (mFI-5). Body composition measures included CT-derived skeletal muscle index (SMI) and density (SMD). Systemic inflammatory status was determined using Systemic Inflammatory Grade (SIG). Outcomes of interest were the incidence of post-operative complications and thirty-day mortality. Associations between categorical variables were examined using χ^2^ test and binary logistics regression analysis.

**Results:**

1002 patients met the inclusion criteria. 28% (*n* = 221) scored 2 or more on the mFI-5. 39% (*n* = 388) of patients had a post-operative complication (Clavien-Dindo I-IV) and 1% (*n* = 11) died within thirty days of surgery. On univariate analysis, mFI-5 frailty score, was significantly associated with advanced age (*p* < 0.001), colonic tumours (*p* < 0.001), reduced use of neo-adjuvant chemotherapy (*p* < 0.05), higher BMI (*p* < 0.05), low SMD (*p* < 0.001), elevated NLR (*p* < 0.05), elevated mGPS (*p* < 0.05), elevated SIG (*p* < 0.05), incidence of post-operative complications (*p* < 0.001) and thirty-day mortality (*p* < 0.05). On multivariate analysis, male sex (*p* < 0.05), elevated SIG (*p* < 0.05) and mFI-5 score (*p* < 0.01) remained significantly associated with the incidence of post-operative complications. mFI-5 frailty was found to remain significantly associated with the incidence post-operative complications in patients who were SIG 0 (*p* < 0.05).

**Conclusion:**

mFI-5 frailty score was found to be significantly associated with age, systemic inflammation and post-operative outcomes in patients undergoing potentially curative resections for CRC. Incorporation of an assessment of systemic inflammatory status in future frailty screening tools may improve their prognostic value.

**Supplementary Information:**

The online version contains supplementary material available at 10.1186/s12877-022-03703-2.

## Introduction

Frailty is a complex multifactorial syndrome, characterised by a clinically significant increase in vulnerability of the patient and worsened health outcomes [1]. Frailty is considered to represent the systemic burden of human aging and erosion of a patient’s homeostatic reserve [2], it remains a growing area of interest in many subspecialities of medicine. Particularly, determining likely outcomes in older adults undergoing surgery [3–5].

In the U.K., over a third of newly diagnosed colorectal cancers (CRC) are in patients aged 75 years and older [6]. As such, the prognostic value of frailty screening measures to clinical outcomes in patients undergoing surgery for CRC has been widely examined [7–9]. One such example is the American College of Surgeons National Surgical Quality Improvement Programs (ACS NSQIP) five-item modified frailty index (mFI-5 [10]. Scores were calculated on the presence of co-morbid disease and non-independent functional status, with increased mFI-5 scores associated with the incidence of post-operative complications and thirty-day mortality in older adults undergoing surgery for CRC [11].

While the current literature suggests an association with frailty and clinical outcomes in patients undergoing surgery for CRC [7, 11], the basis of relationship is unclear. Indeed, frailty been associated with prognostic, pre-operative host factors including malnutrition, sarcopenia and inflammation [12–16]. However, there is a paucity of research examining the association between frailty and such factors in CRC [17]. Therefore, the aim of the present study was to examine the relationship between frailty, screened for using the mFI-5 frailty tool, malnutrition, body composition, systemic inflammation and short-term clinical outcomes in patients undergoing surgery for colorectal cancer.

## Patients and methods

Retrospective analysis of prospectively collected data from consecutive patients who underwent potentially curative resection for colorectal cancer, at Glasgow Royal Infirmary, between April 2008 and April 2018 was carried out. Patients who had electronic medical records facilitating calculation of the mFI-5, pre-operative CT imaging, recorded height and weight, pre-operative assessment of systemic inflammatory status and had TNM stage I-III disease were assessed for inclusion. Exclusion criteria were as follows; patients whose medical records did not facilitate calculation of mFI-5 score, patients without satisfactory pre-operative CT imaging, patients without a recorded height and weight, patients who had no pre-operative assessment of the systemic inflammatory or had TNM Stage IV disease. Ethical approval from the West of Scotland Ethics Committee, Glasgow was granted to collect such routine clinicopathological data. Written informed consent for each patient was obtained prior to surgery for the collection of routine clinicopathological details. There are no patient identifiable details included requiring consent. The study was conducted in accordance with the Declaration of Helsinki and conformed to the STROBE guidelines for cohort studies.

The primary outcomes of interest were the incidence of post-operative complications and thirty-day mortality.

All patients were operated on at a single centre. A proportion of patients, primarily those with rectal tumours, received neo-adjuvant chemotherapy. Pre-operatively, all patients received thromboembolism and antibiotic prophylaxis as per local protocols. Perioperative care was standardized using an enhanced recovery protocol. Postoperatively, all patients underwent daily clinical assessment by a member of the surgical team. Additional investigations and management were instigated at the discretion of the surgical team based on the relevant clinical findings, as previously described [18]. The incidence of post-operative complications was prospectively recorded using the Clavien-Dindo classification [19] and patients categorised as complication/no complication. The incidence of thirty-day mortality was also prospectively recorded.

### Clinico-pathological characteristics

Routine demographic details included age, sex and BMI. Age categories were grouped into < 64, 65–74 and > 74 years. Tumour site was identified from pre-operative CT imaging, endoscopic and pathology reports. Tumours were staged using the fifth edition of the TNM classification, consistent with practice current in the United Kingdom during the study period [20]. The Malnutrition Universal Screening Tool (MUST) was used to determine the overall risk of malnutrition. MUST is a 3-component score consisting of the patient’s current weight status using BMI, unintentional weight loss, and the acute disease effect. Scores were identified retrospectively from patient’s medical records. Assessment was made by clinical nursing staff, using a dedicated proforma, as previously described [21]. BMI was categorised as < 20, 20-24.9, 25-29.9 and ≥ 30 kg/m^2^.

### Frailty

Frailty was defined using the modified five-item frailty index (mFI-5), developed from the American College of Surgeons National Surgical Quality Improvement Program (ACS NSQIP, [22]. The mFI-5 screening tool combines both functional status and co-morbidity and is scored from 0 to 5. Patients are allocated 1 point for each of the following criterion- congestive heart failure, chronic obstructive pulmonary disease (COPD) or recent pneumonia, hypertension requiring medication, diabetes mellitus and non-independent functional status. The presence of co-morbid disease and functional status of patients were retrospectively identified from pre-operative anaesthetic assessments and medical records. Non-independent functional status was defined as the patient either partially or totally dependent prior to surgery. This was determined by patients having an Eastern Cooperative Oncology Group (ECOG) performance status of ≥ 2, having a documented history of requiring a package of care or a history of a relative acting as a carer for the patient. Patients scores were grouped as 0/1/≥2.

### CT-derived Body composition

CT images were obtained at the level of the third lumbar vertebra, as previously described [23]. Patients with CT imaging taken 3 months or more prior to their surgery were excluded from the study. Furthermore, scans with significant movement artefact or missing regions of interest were not considered for inclusion. Each image was analysed using a free-ware program (NIH Image J version 1.47, http://rsbweb.nih.gov/ij/) shown to provide reliable measurements [24].

Region of interest (ROI) measurements were made of the total fat area (TFA, cm^2^), visceral fat area (VFA, cm^2^) and skeletal muscle area (SMA, cm^2^) using standard Hounsfield Unit (HU) ranges (adipose tissue − 190 to -30, and skeletal muscle − 29 to + 150). The VFA was then subtracted from the TFA to calculate the subcutaneous fat area (SFA, cm^2^). The SFA and SMA were then normalised for height^2^ to create indices; subcutaneous fat index (SFI, cm^2^/m^2^), and skeletal muscle index (SMI, cm^2^/m^2^). Skeletal muscle radiodensity (SMD, HU) was measured from the same ROI used to calculate SMI, as its mean HU [23]. These indices were then compared with established thresholds for body composition status [25–27].

### Systemic inflammation

Pre-operative haematological and biochemical results were identified from medical records and prospectively recorded. Blood samples were either obtained at pre-operative assessment, within 30 days of surgery, for elective patients or on admission for patients undergoing emergency surgery, as previously described [28]. An autoanalyzer was used to measure serum CRP (mg/L) and albumin (g/L) concentrations (Architect; Abbot Diagnostics, Maidenhead, UK).

Systemic inflammatory status was retrospectively assessed by calculating the neutrophil/lymphocyte ratio (NLR) and modified Glasgow Prognostic Score (mGPS) for each patient, using pre-operative blood results. The NLR was calculated by division of the was neutrophil count by the lymphocyte count, obtained from the patient’s full blood count (FBC). NLR values were grouped as < 3 (considered normal), 3–5 (considered moderate), and > 5 (considered raised), as previously described [24]. The mGPS was derived as the following: patients with a normal CRP (< 10 mg/L) scored 0, those with an elevated CRP (> 10 mg/L) alone were scored 1, and those with an elevated CRP (> 10 mg/L) and hypoalbuminemia (< 35 g/L) were scored 2, as previously described [29].

The NLR and mGPS, were also combined to form the Systemic Inflammatory Grade (SIG), as previously described [30]. Patients were categorised as grade 0–4, as follows: SIG 0 was defined as mGPS 0 and NLR < 3; SIG 1 as mGPS 0 and NLR 3–5 or mGPS 1 and NLR < 3; SIG 2 as mGPS 0 and NLR > 5 or mGPS 2 and NLR < 3 or mGPS 1 and NLR 3–5; SIG 3 as mGPS 1 and NLR > 5 or mGPS 2 and NLR 3–5 and SIG 4 as mGPS 2 and NLR > 5 (see Table 1).

**Table 1:** Prevalence of frailty screening items of the mFI-5 of included patients (*n* = 1002)

Item	Patients (*n *= /%)
Congestive Heart Failure	21 (2%)
Chronic Obstructive Pulmonary Disease or recent pneumonia	66 (7%)
Hypertension (Requiring medication)	451 (45%)
Diabetes Mellitus	151 (15%)
Non-independent Functional Status	184 (18%)

### Statistical analysis

Demographic data, clinicopathological variables, mFI-5, MUST, BMI, CT-body composition measures, NLR, mGPS, SIG, incidence of post-operative complication and thirty-day mortality were presented as categorical variables. The Pearson Chi square test was used to examine the associations between categorical variables and the Chi square test for linear trend was used for ordered variables with multiple categories.

Associations between mFI-5 frailty score, demographic data, clinicopathological variables, MUST, BMI, CT-body composition measures, SIG, incidence of post-operative complication and thirty-day mortality were analysed using univariate and multivariate binary logistics regression analysis with a backward conditional approach. A *p* < 0.1 was applied to inclusion at each step in the multivariate analysis.

Missing data were excluded from analysis on a variable-by-variable basis. Two-tailed *p* values < 0.05 were considered statistically significant. Statistical analysis was performed using SPSS software version 25.0. (SPSS Inc., Chicago, IL, USA).

## Results

In total, 1002 patients met the inclusion criteria. 55% (*n* = 554) of patients were male and 66% (*n* = 657) were aged 65 years or older. 35% (*n* = 350) of patients were ASA ≥ 3. 60% (*n* = 602) of patients had colonic tumours and 40% (*n* = 400) had rectal. 24% (*n* = 240) of patients had TNM stage I disease, 40% (*n* = 404) stage II and 36% (*n* = 358) stage III. 14% (*n* = 138) of patients received neo-adjuvant chemotherapy. 18% (*n* = 174) of those with a pre-operative MUST were at risk of malnutrition (MUST ≥ 1). The median BMI of the cohort was 27 kg/m2 and 65% (*n* = 652) of patients had a BMI ≥ 25 kg/m2. A high VFA was present in 73% (*n* = 731) of patients and 80% (*n* = 803) had a high SFI. A low SMI and SMD were present in 57% (*n* = 570) and 58% (*n* = 584), respectively. 48% (*n* = 479) of patients had an NLR ≥ 3 and 27% (*n* = 271) had an mGPS ≥ 1. 43% (*n* = 427) of patients were SIG 0, 26% (260) SIG 1 and 31% (*n* = 315) were SIG ≥ 2. 39% (*n* = 388) had a post-operative complication (Clavien-Dindo I-IV). 1% (*n* = 11) of patients died within thirty days of surgery.

The prevalence of mFI-5 frailty screening items of included patients is shown in Table 1. 2% (*n* = 21) of patients had congestive heart failure, 7% (*n* = 66) had COPD or recent pneumonia, 45% (*n* = 451) had hypertension requiring medication, 15% (*n* = 151) had diabetes mellitus and 18% (*n* = 184) had non-independent functional status. The prevalence of mFI-5 frailty scores of included patients is shown in Table 2. 40% (*n* = 397) of patients scored 0 using mFI-5 frailty screening tool, 38% (*n* = 384) scored 1, 28% (*n* = 221) scored 2 or more.

**Table 2:** The mFI-5 frailty scores of included patients (*n* = 1002)

mFI-5 Frailty Score	Patients (*n *= /%)
0	397 (40%)
1	384 (38%)
2	180 (18%)
3	36 (4%)
≥ 4	5 (< 1%)

The relationship between clinicopathological variables, malnutrition, CT-BC measurements, systemic inflammation, length of stay and incidence of post-operative complications in patients undergoing potentially curative resection for colorectal cancer, stratified by mFI-5 frailty, is shown in Table 3. On univariate analysis, mFI-5 frailty score, was significantly associated with advanced age (*p* < 0.001), colonic tumours (*p* < 0.001), reduced use of neo-adjuvant chemotherapy (*p* < 0.05), higher BMI (*p* < 0.05), low SMD (*p* < 0.001), elevated NLR (*p* < 0.05), elevated mGPS (*p* < 0.05), elevated SIG (*p* < 0.05), incidence of post-operative complications (*p* < 0.001) and thirty-day mortality (*p* < 0.05). Frailty was not associated with sex (*p* = 0.697), TNM stage (*p* = 0.072), MUST risk (*p* = 0.630), high SFI (*p* = 0.299)   or low SMI (*p* = 0.407).

**Table 3:** The relationship between clinicopathological variables, malnutrition, CT-BC measurements and systemic inflammation in patients undergoing potentially curative resection for CRC, stratified by mFI-5 frailty score (*n* = 1002)

	mFI-5= 0 (*n*=397)/%	mFI-5 = 1 (*n* = 384) /%	mFI-5 ≥ 2 (*n* = 221)/ %	***P*** Value^1^
**Age (years)**				
< 65	165 (42%)	114 (30%)	66 (30%)	< 0.001
65-74	157 (40%)	135 (35%)	75 (34%)	
>74	75 (19%)	135 (35%)	80 (36%)	
**Sex**				
Male	222 (56%)	212 (55%)	120 (54%)	0.697
Female	175 (44%)	172 (45%)	101 (46%)	
**Tumour Site**				
Colon	209 (53%)	246 (64%)	147 (67%)	< 0.001
Rectum	188 (47%)	138 (36%)	74 (34%)	
**TNM Stage**				
I	89 (23%)	98 (26%)	53 (24%)	0.072
II	148 (37%)	158 (41%)	98 (44%)	
III	160 (40%)	138 (33%)	70 (32%)	
**Neo-adjuvant chemotherapy**				
No	325 (83%)	338 (88%)	194 (89%)	0.024
Yes	68 (17%)	45 (12%)	25 (11%)	
**MUST Risk** ^**2**^				
Low	331 (84%)	302 (81%)	177 (82%)	0.630
Medium	30 (8%)	36 (10%)	25 (12%)	
High	32 (8%)	36 (10%)	15 (7%)	
**BMI (kg/m** ^**2**^ **)**				
< 20	23 (6%)	28 (7%)	7 (3%)	0.034
20-24.9	114 (29%)	122 (32%)	56 (25%)	
25-29.9	147 (37%)	118 (31%)	72 (33%)	
≥ 30	113 (29%)	116 (30%)	86 (39%)	
**High SFI**				
No	81 (20%)	82 (21%)	36 (16%)	0.299
Yes	316 (80%)	302 (79%)	185 (84%)	
**High VFA**				
No	111 (28%)	113 (29%)	47 (21%)	0.128
Yes	286 (72%)	271 (71%)	174 (79%)	
**Low SMI**				
No	204 (51%)	184 (48%)	104 (48%)	0.407
Yes	193 (49%)	200 (52%)	114 (52%)	
**Low SMD**				
No	163 (41%)	112 (29%)	59 (27%)	< 0.001
Yes	234 (59%)	272 (71%)	162 (73%)	
**NLR**				
< 3	224 (56%)	194 (51%)	105 (48%)	0.019
3-5	116 (29%)	121 (31%)	73 (33%)	
> 5	57 (14%)	69 (18%)	43 (19%)	
**mGPS**				
0	301 (76%)	271 (70%)	159 (72%)	0.028
1	50 (13%)	41 (11%)	18 (8%)	
2	46 (12%)	72 (19%)	44 (20%)	
**SIG**				
0	187 (47%)	155 (40%)	85 (39%)	0.006
1	107 (27%)	94 (25%)	59 (27%)	
2	58 (15%)	80 (21%)	41 (19%)	
≥ 3	45 (11%)	55 (14%)	36 (16%)	
**Post Operative Complication**				
No	274 (69%)	214 (56%)	126 (57%)	< 0.001
Yes	123 (31%)	170 (44%)	95 (43%)	
**Thirty-day Mortality**				
No	395 (99%)	381 (99%)	215 (97%)	0.019
Yes	2 (1%)	3 (1%)	6 (3%)	

^1^*P* value from χ2 analysis

^2^ 18 patients missing MUST assessment

The relationship between clinicopathological variables, mFI-5 frailty score, malnutrition, CT-BC measurements, systemic inflammation and incidence of post-operative complications in patients undergoing potentially curative resection for colorectal cancer is shown in Table 4. On multivariate analysis, male sex (*p* < 0.05), elevated SIG (*p* < 0.05) and mFI-5 frailty score (*p* < 0.01) remained significantly associated with the incidence of post-operative complications.

**Table 4:** The relationship between clinicopathological variables, BMI, CT-BC measurements, systemic inflammation, frailty and the incidence of post-operative complications in patients undergoing potentially curative resection for CRC (*n* = 1002)

	OR (Univariate)	*P* Value	OR (Multivariate)	*P* Value
Age (< 65/65–74/>74)	1.28 (1.00-1.38)	0.047	-	0.241
Sex (Male/Female)	1.40 (1.08–1.81)	0.011	1.39 (1.07–1.80)	0.013
Tumour Site (Colon/Rectum)	1.13 (0.87–1.47)	0.347	-	-
Neo-adjuvant chemotherapy (No/Yes)	1.10 (0.76–1.58)	0.624	-	-
MUST risk (Low/Medium/ High risk)	1.08 (0.87–1.33)	0.498	-	-
BMI (< 20/20-24.9/25-29.9/≥30)	1.03 (0.90–1.19)	0.668	-	-
High SFI (No/Yes)	1.09 (0.79–1.50)	0.619	-	-
High VFA (No/Yes)	1.07 (0.80–1.42)	0.668	-	-
Low SMI (No/Yes)	1.10 (0.85–1.42)	0.462	-	-
Low SMD (No/Yes)	1.13 (0.86–1.48)	0.384	-	-
SIG (0/1/2/≥3)	1.17 (1.05–1.30)	0.004	1.14 (1.03–1.27)	0.014
mFI-5 Score (0/1/≥2)	1.33 (1.13–1.58)	< 0.001	1.32 (1.11–1.56)	0.001

The relationship between mFI-5 frailty score, systemic inflammation and incidence of post-operative complications in patients undergoing potentially curative resection for colorectal cancer is shown in Table 5. On univariate analysis, SIG was associated with the incidence of post-operative complications (*p* < 0.05). On univariate analysis, mFI-5 frailty score was significantly associated with the incidence of post-operative complications (*p* < 0.05). In patients who were not inflamed (SIG 0), mFI-5 frailty score was significantly associated with the incidence of post-operative complications (*p* < 0.05). In patients who were mFI-5 0, SIG was not associated with the incidence of post-operative complications (*p* = 0.243).

**Table 5:** The relationship between mFI-5 frailty score, systemic inflammation and incidence of post-operative complications in patients undergoing potentially curative resection for CRC (*n* = 1002)

	mFI-5 0 (*n* = 397)	mFI-5 1 (*n* = 384)	mFI-5 ≥ 2 (*n* = 221)	mFI-5 (0-≥2)	*P* Value
**SIG 0 (** ***n*** ** = 427)**	51 (27%)	62 (40%)	33 (39%)	146 (34%)	0.024
**SIG 1 (** ***n*** ** = 260)**	36 (34%)	44 (47%)	26 (44%)	106 (41%)	0.121
**SIG 2 (** ***n*** ** = 179)**	22 (38%)	32 (40%)	17 (42%)	71 (40%)	0.719
**SIG ≥ 3 (** ***n*** ** = 136)**	14 (31%)	32 (58%)	19 (53%)	65 (48%)	0.039
**SIG (0-≥3)**	123 (31%)	170 (44%)	95 (43%)	388 (39%)	0.006
***P*** **Value**	0.243	0.080	0.219	0.001	

In each cell complication incidence (%)

## Discussion

The results of the present study showed that, in a large cohort of patients undergoing potentially curative surgery for colorectal cancer, the mFI-5 frailty score was found to be associated with age, tumour site, neo-adjuvant chemotherapy, BMI, SMD, NLR, mGPS, SIG the incidence of post-operative complications and thirty-day mortality. However, mFI-5 and SIG were independently associated with the incidence of post-operative complications. Therefore, the mFI-5 index has clinical utility and would appear to capture the prognostic impact of some elements of nutritional status on the incidence of post-operative complications, but not that of the systemic inflammatory response.

In the present study, the mFI-5 frailty score was found to be significantly associated with systemic inflammation and the incidence of post-operative complications, even in patients younger than 65 years of age (see Supplementary Tables 1 and 2). While an association between frailty and short-term outcomes (incidence of post-operative complications, length of stay and thirty-day mortality) has been widely reported in patients undergoing surgery for CRC, the basis of this relationship remains unclear. It has been postulated that an exaggerated systemic inflammatory response may be responsible for adverse clinical outcomes in frail patients [11]. Indeed, Soysal and co-workers reported an association between frailty and systemic inflammation in a recent systematic review and meta-analysis, in keeping with the present observations [14]. However, frailty was found to remain significantly associated with the incidence of post-operative complications in patients who were not inflamed (SIG 0, See Table 5). As such, the relationship between frailty, systemic inflammation and short-term outcomes in patients undergoing surgery for CRC remains unclear and requires further study.

Frailty is thought to encompass not only age, but a number of recognised domains including functional status, malnutrition, co-morbidity, cognition, socio-economic and psychological factors [31, 32]. Indeed, the present study that found mFI-5 frailty scores were significantly associated with short-term clinical outcomes, even when younger patients (< 65 years) were studied in insolation (see Supplementary Table 1). The present observations are in keeping with those of Miller and co-workers, who in a cohort of 9, 252 patients undergoing proctectomy for CRC, found that frailty, but not age, was associated with adverse post-operative outcomes [33]. Taken together, these results that such frailty screening measures may have prognostic value in younger adults undergoing surgery for CRC, as an assessment of their robustness to the physiological stress of surgery. Furthermore, that simply screening for frailty in patients of advanced age is insufficient and that those who are functionally restricted or co-morbid are also likely to be frail and at increased risk of adverse outcomes following surgery.

In the present study it was of interest that frailty, determined using the mFI-5 frailty score, was not associated with recognised prognostic host factors in CRC including malnutrition and low skeletal muscle mass [21, 34]. Indeed, a loss of skeletal muscle mass is one of many causes of functional impairment, a hallmark of frailty [35]. Furthermore, malnutrition has been shown to be prevalent in frail, older adults [13, 36]. However, since frailty screening tools may capture many elements of ageing including nutritional status, performance status, cognitive status and now from this work inflammatory status, it is likely that the contribution of these elements to a high frailty score will vary with the disease condition. Therefore, although mFI-5 is a convenient screening tool it is important to define which element is the main driver of the frailty score so that this may be targeted in the patient. Specifically, if the present results are confirmed, frailty screening measures should be utilised in combination with other recognised prognostic host-assessments such as MUST, CT-derived body composition and systemic inflammatory status in patients undergoing surgery for CRC [21, 30, 34].

There are a number of limitations to the present study. Firstly, the study was retrospective in nature and subject to sample bias. Specifically, the retrospective scoring of frailty using the mFI-5. However, in the present cohort, around 22% (*n* = 227) of patients undergoing surgery for CRC had an mFI-5 ≥ 2. This is in keeping with the observations of Al-Khamis and co-workers, who found a similar prevalence of mFI-5 score of ≥ 2 (18%) in a cohort of 295, 490 patients undergoing colorectal surgery [37]. As such the present observations are likely to be reliable. Secondly, functional impairment is a recognised hallmark of frailty and there is no objective measure of functional status utilised in the mFI-5. As such, there is potential for significant variation in the level of physical function in patients deemed to have non-independent functional status. Associations between routine measures of physical function and the mFI-5 frailty index will therefore be informative. Lastly, the present study included only patients who underwent surgical resection with curative intent and not those with advanced or metastatic cancer. Further studies of frailty across other cancer subtypes and disease stages will be required to delineate the prognostic value of the mFI-5 frailty index to cancer outcomes.

In conclusion, mFI-5 frailty score was found to be significantly associated with age, systemic inflammation and post-operative outcomes in patients undergoing potentially curative resections for CRC. Incorporation of an assessment of systemic inflammatory status in future frailty screening tools may improve their prognostic value.

## Supplementary Information

**Additional file 1.**

**Additional file 2.**

## Data Availability

The datasets generated and/or analysed during the current study are not publicly available but are available from the corresponding author on reasonable request.
