# Supplementary material for: The relationship between the modified frailty index score (mFI-5), malnutrition, body composition, systemic inflammation and short-term clinical outcomes in patients undergoing surgery for colorectal cancer
Source: BMC Geriatr. 2023 Jan 6;23:9. doi: 10.1186/s12877-022-03703-2 (PMC9817261; doi:10.1186/s12877-022-03703-2)
Supplement: Supplementary file 1 — Additional file 1. [file 12877_2022_3703_MOESM1_ESM.docx]

**Supplementary Table 1.** The relationship between clinicopathological variables, malnutrition, CT-BC measurements, systemic inflammation, length of stay and incidence of post-operative complications in patients younger than 65 years of age, undergoing potentially curative resection for CRC, stratified by mFI-5 frailty (n=345).

|  | **mFI-5= 0**  (n=165)/ % | **mFI-5= 1**  (n=114) / % | **mFI-5 ≥2**  (n=66)/ % | **P Value^1^** |
| --- | --- | --- | --- | --- |
| **Sex**  Male  Female | 84 (51%)  81 (49%) | 60 (53%)  54 (47%) | 33 (50%)  33 (50%) | 0.977 |
| **Tumour Site**  Colon  Rectum | 79 (48%)  86 (52%) | 61 (54%)  53 (46%) | 34 (52%)  32 (48%) | 0.489 |
| **TNM Stage**  I  II  III | 36 (22%)  55 (33%)  74 (45%) | 33 (29%)  40 (35%)  41 (36%) | 17 (26%)  27 (41%)  22 (33%) | 0.103 |
| **Neo-adjuvant chemotherapy**  No  Yes | 135 (82%)  30 (18%) | 93 (82%)  21 (18%) | 52 (79%)  14 (21%) | 0.634 |
| **MUST Risk^2^**  Low  Medium  High | 143 (87%)  11 (7%)  10 (6%) | 100 (89%)  6 (5%)  6 (5%) | 55 (83%)  6 (9%)  5 (8%) | 0.628 |
| **BMI (kg/m^2^)**  <20  20-24.9  25-29.9  ≥30 | 9 (6%)  43 (26%)  63 (38%)  50 (30%) | 6 (5%)  30 (26%)  35 (31%)  43 (38%) | 2 (3%)  15 (23%)  17 (26%)  32 (49%) | 0.503 |
| **High SFI**  No  Yes | 35 (21%)  130 (80%) | 21 (18%)  93 (82%) | 12 (18%)  54 (82%) | 0.539 |
| **High VFA**  No  Yes | 56 (34%)  109 (66%) | 36 (32%)  78 (68%) | 14 (21%)  52 (79%) | 0.078 |
| **Low SMI**  No  Yes | 97 (59%)  68 (41%) | 76 (67%)  38 (33%) | 44 (67%)  22 (33%) | 0.178 |
| **Low SMD**  No  Yes | 83 (50%)  82 (50%) | 60 (53%)  54 (47%) | 32 (49%)  34 (52%) | 0.913 |
| **NLR**  <3  3-5  >5 | 104 (63%)  36 (22%)  25 (15%) | 60 (53%)  38 (33%)  16 (14%) | 29 (44%)  25 (38%)  12 918%) | 0.039 |
| **mGPS**  0  1  2 | 128 (78%)  23 (14%)  14 (8%) | 84 (74%)  16 (14%)  14 (12%) | 47 (71%)  7 (11%)  12 (18%) | 0.096 |
| **SIG**  0  1  2  ≥3 | 87 (53%)  36 (22%)  27 (16%)  14 (9%) | 53 (47%)  26 (23%)  20 (18%)  15 (13%) | 26 (39%)  18 (27%)  10 (15%)  12 (18%) | 0.032 |
| **Post Operative Complication**  No  Yes | 118 (72%)  47 (29%) | 67 (59%)  47 (41%) | 38 (58%)  28 (42%) | 0.019 |
| **Thirty-day Mortality**  No  Yes | 164 (99)  1 (1) | 114 (100)  0 (0) | 65 (88)  1 (2) | 0.596 |

*^1^ P value from χ2 analysis*

*^2^ 3 patients missing MUST assessment.*
