# Supplementary material for: The relationship between the modified frailty index score (mFI-5), malnutrition, body composition, systemic inflammation and short-term clinical outcomes in patients undergoing surgery for colorectal cancer
Source: BMC Geriatr. 2023 Jan 6;23:9. doi: 10.1186/s12877-022-03703-2 (PMC9817261; doi:10.1186/s12877-022-03703-2)
Supplement: Supplementary file 2 — Additional file 2. [file 12877_2022_3703_MOESM2_ESM.docx]

**Supplementary Table 2**. The relationship between clinicopathological variables, BMI, CT-BC measurements, systemic inflammation, frailty and the incidence of post-operative complications in patients younger than 65 years of age, undergoing potentially curative resection for CRC (n=345).

|  | **OR (Univariate)** | **P Value** | **OR (Multivariate)** | **P Value** |
| --- | --- | --- | --- | --- |
| Sex (Male/Female) | 1.13 (0.73-1.76) | 0.587 | - | - |
| Tumour Site (Colon/Rectum) | 1.39 (0.89-2.17) | 0.142 | - | - |
| Neo-adjuvant chemotherapy (No/Yes) | 1.18 (0.68-2.06) | 0.562 | - | - |
| MUST Risk (Low/Medium/ High risk) | 1.62 (1.08-2.44) | 0.021 | 1.61 (1.07-2.43) | 0.023 |
| BMI (<20/20-24.9/25-29.9/≥30) | 1.05 (0.82-1.34) | 0.714 | - | - |
| High SFI (No/Yes) | 1.09 (0.62-1.90) | 0.767 | - | - |
| High VFA (No/Yes) | 1.09 (0.68-1.77) | 0.717 | - | - |
| Low SMI (No/Yes) | 1.10 (0.70-1.73) | 0.686 | - | - |
| Low SMD (No/Yes) | 0.99 (0.64-1.55) | 0.979 | - | - |
| SIG (0/1/2/≥3) | 1.22 (1.01-1.48) | 0.040 | - | 0.142 |
| mFI-5 Score (0/1/≥2) | 1.41 (1.06-1.88) | 0.019 | 1.43 (1.07-1.91) | 0.017 |
